# Inferring time-dependent population growth rates in cell cultures undergoing adaptation

**DOI:** 10.1186/s12859-020-03887-7

**Published:** 2020-12-17

**Authors:** H. Jonathan G. Lindström, Ran Friedman

**Affiliations:** Department of Chemistry and Biomedical Sciences, Linnaeus University, 391 82 Kalmar, Sweden

**Keywords:** Growth rate, Adaptation, Cell counting

## Abstract

**Background:**

The population growth rate is an important characteristic of any cell culture. During sustained experiments, the growth rate may vary due to competition or adaptation. For instance, in presence of a toxin or a drug, an increasing growth rate indicates that the cells adapt and become resistant. Consequently, time-dependent growth rates are fundamental to follow on the adaptation of cells to a changing evolutionary landscape. However, as there are no tools to calculate the time-dependent growth rate directly by cell counting, it is common to use only end point measurements of growth rather than tracking the growth rate continuously.

**Results:**

We present a computer program for inferring the growth rate over time in suspension cells using nothing but cell counts, which can be measured non-destructively. The program was tested on simulated and experimental data. Changes were observed in the initial and absolute growth rates, betraying resistance and adaptation.

**Conclusions:**

For experiments where adaptation is expected to occur over a longer time, our method provides a means of tracking growth rates using data that is normally collected anyhow for monitoring purposes. The program and its documentation are freely available at https://github.com/Sandalmoth/ratrack under the permissive zlib license.

## Background

Population growth rates are a core property of cell lines, and can be influenced by many factors. As such, in controlled experiments, cell growth rates, i.e. how quickly the population size changes over time, may correlate with the presence of drugs or toxins [1], temperature [2], or particular genetic changes in the cells [3], and other factors. The cell growth rate is an especially useful variable to feed into models of how a culture will progress [4–6]. Whereas well established mathematical definitions of the growth rate exist [7], it is not obvious how to estimate the growth rate from measurements of cell cultures. Suspension cells follow logistic growth to a decent approximation, and in that case growth rate could be derived via some form of logistic fitting.

The most common measurement for cell culture growth rate is the so-called population doubling time (PDT), i.e. the time it takes for a population to double its size [8]. The doubling time can be estimated from the population size at two points [8]. For cells growing exponentially this value is well-defined. However, the more a cell culture strays from exponential growth, the more inaccurate the PDT becomes a measure of population cell growth. It is no longer consistently applicable starting from any time. Exponential growth is a consequence of growth laws in individual cells causing regular division [9, 10]; the addition of factors such as cooperation or competition for resources creates a non-exponential population growth.

Under a different model of population growth, such as logistic growth, it is possible to separate the idea of growth rate from the decrease as the population approaches its carrying capacity. There are other approaches towards finding these growth parameters based on various methods, e.g. steepest descent optimization [11], and different means of Bayesian inference [12, 13]. Some models use highly detailed representations of the cell cycle [14]. However, these models generally do not consider a changing growth rate, and focus on inferring a static growth rate and carrying capacity. In reality, the growth rate sometimes changes over time, either because of density-dependent effects or because the cells are evolving. Examples of processes that result in modifications to the growth rate include adaptation to the presence of drugs [15, 16] and to lack of nutrients [17]. In general, any change in the evolutionary landscape might lead to a modification the growth rate.

Almost all available cell counting methods require fairly large sample sizes ($> 10^5$ cells or $> 1$ mL) [18]. Haemocytometer counting with some vital stain stands out as an exception, as it requires only a very minimal sample (5–10 $\upmu \,\hbox {L}$, typically $< 10^4$ cells). For monitoring a culture, especially in small volumes, requiring a large sample can be a limiting factor. Owing to its low cost and availability, haemocytometer counting has become the de facto standard in many applications. That, coupled with being measurable without significantly affecting an ongoing culture, makes it a prime choice for estimating the population growth rate.

The population size, i.e., the number of living cells, is inextricably tied to the growth rates, as the growth rate describes the positive component of the rate of change in population size. There is typically some basal death rate as well, although it is often difficult to separate apparent growth rate into the true growth rate and death rate. Unlike the population size, growth rate is not an observable variable and has to be inferred. By assuming a model of how cells grow, the growth rate can be deduced from the population size, which can be measured in ways that hardly affect the cell culture. Thus, it should be possible to track the (time dependent) growth rate in a cell culture over longer periods of time.

We developed a method to calculate the growth rates in a suspended cell lines. To use our approach, it is necessary to know the carrying capacity beforehand, but in return the growth rate is allowed to change over time. Our program is in principle agnostic to the type of measurement chosen, so long as it is proportional to the number of viable cells. However, it is structured primarily to work with cell counts (collected manually or by machine). Given the generality of the approach, it is possible to work with other measures as well, including optical densities, measurements of metabolites, etc.

## Implementation

Cells in suspension culture typically follow logistic growth to a reasonable approximation. Logistic growth can be broken down into two elements: a given rate of division per cell, and a death rate that depends linearly on the population size [19]. Such a death-rate is equivalent to pairwise negative interactions between all cells in the population. Any genetic change or response in the cells might modify either of these parameters. We limit our model to changes in the growth rate, since death rates and the carrying capacity are coupled to nutrient availability, and are hence assumed to stay rather constant in any cell line.

The growth rate *r*(*t*) is represented as a piecewise linear function with evenly distributed segment lengths. This simple representation was chosen to reduce the risk of overfitting, as it requires as few as two (or even one, for a constant growth rate) parameters. The number of cells at any particular time, *N*(*t*), with carrying capacity *K*, can be obtained from

1 $$\begin{aligned} \frac{dN}{dt} = r(t) N - r(t) \frac{N^2}{K} \end{aligned}$$

which we arrive at by substituting a time-dependent growth rate into a standard logistic growth model [7]. Solving this Bernoulli differential equation [20] yields

2 $$\begin{aligned} N(t) = \frac{\exp \left[ \int _0^t r(\xi ) d\xi \right] }{N_0 + \int _0^t \frac{r(\zeta )}{K} \exp \left[ \int _0^\zeta r(\xi ) d\xi \right] d\zeta } . \end{aligned}$$

Alternatively, the population curve can be obtained by simulating a logistic branching process [19], which is slower but likely more realistic, especially when the population size is low. In such a process, cells are modelled as individual particles that divide after some randomly distributed length of time. It is a type of model that very obviously connects to the reality of the situation. So, by extending the model in [19] with a time-dependent growth rate, in the stochastic simulations, the growth rate is given by

3 $$\begin{aligned} \lambda = Nr(t) \end{aligned}$$

and the death rate by

4 $$\begin{aligned} \mu = N(N - 1)\frac{r(t)}{K} \end{aligned}$$

which is then simulated using the next reaction method [21]. Both simulation methods have been implemented; the stochastic simulator is recommended only for population sizes below $2 \cdot 10^6$, as it is significantly slower for large populations since division and death events for all cells are simulated individually.

Experimental cell counting produces noisy and sparse timelines of the population size over time (e.g. Additional file 1: Figure S1). The piecewise linear growth rate cannot easily be obtained analytically from such data. To overcome this problem, we complete the population curve model above with imitation experimental noise. Approximate Bayesian computation (ABC) can thereafter be used to numerically estimate the growth rate.

The experimental noise consists of two parts:

1. Sampling noise—the error caused by counting cells in a small sample of the cell culture.
2. Counting noise—the errors induced by inaccurate counting.

The sampling noise is modelled by asserting that for well dispersed cells a sample contains a Poisson–distributed number of cells, thus sampling multiple times involves a nested series of Poisson random variables. This alone is known to underestimate the actual noise [22] as the counting noise is not included. Thus, to estimate the counting noise, we implemented a customizable set of filters, as disparate experimental set-ups may warrant different forms of noise. An easily motivated choice is a normally distributed counting noise, which is (approximately) consistent with each individual count having a certain chance of being incorrect. Dividing the noise into two parts is a pragmatic choice to make changing between counting methods easier, as the manual error rate is approximately known [23], whereas manufacturers of automatic counters commonly provide estimates.

**Fig. 1:**
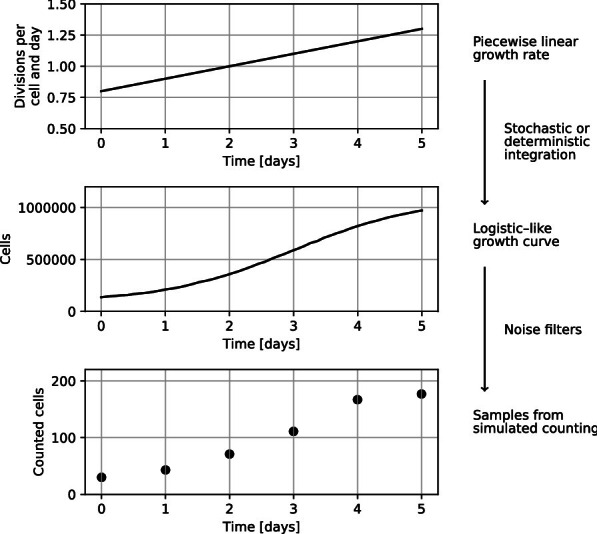
Forward model simulating data that is comparable to experiments. Cell counting is done on a small sample of the total population, hence a small proportional fraction of the total number of cells is expected

This set-up enables simulation from a piecewise linear growth rate over time using samples taken at discrete time points (Fig. 1). This forward model is inverted numerically using pyABC [24] and a sequential Monte-Carlo (SMC) approach. Uniform priors over a user-defined range are used for the growth rate control points. Importantly, the deterministic simulator is well-behaved also for negative growth rates. The output from ABC is samples from the posterior distribution, visualized using connected raincloud plots. We use raincloud plots as they combine a kernel density approximation, a box-plot and raw data plotting into a single figure [25], making it easy to interpret the results.

To summarize, the time-dependent growth rate can be calculated numerically from the following:

1. a series of cell counts.
2. the dilution steps taken for each count.
3. the time when each of the counts was done.
4. an estimate of the carrying capacity.

**1**–**3** are used to estimate the number of cells at a certain time taking measurement error into account, whereas **4** is a fundamental parameter of a certain cell type that has to be provided. This data is processed in a number of steps, which are automatically managed by a workflow system. See the “Usage and practical details” section for a more in depth explanation.

## Results and discussion

### Simulated data example

**Fig. 2:**
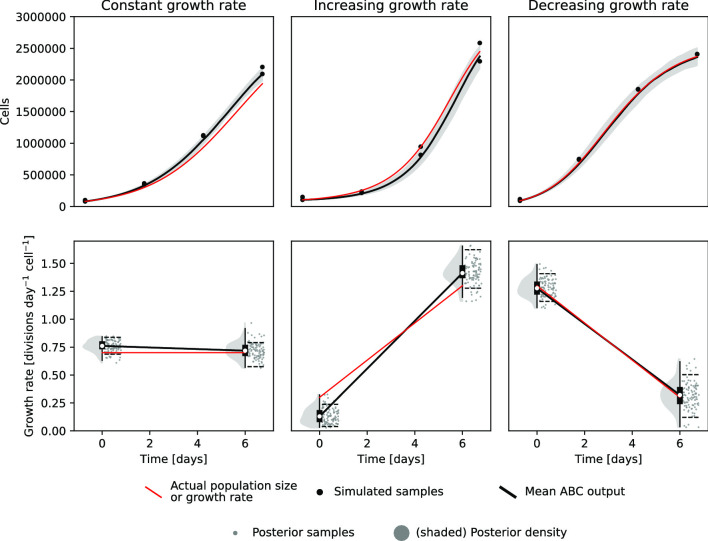
Predicted growth rates from three simulated examples. Cell counting data (black dots, top panels) were generated from population size curves (red lines, top panels) simulated from three different piecewise linear growth rate curves (red lines, bottom panels) using the forward model (Fig. 1). We then then reconstructed the most likely growth rates from the sampled data (black), but, since sampling is inherently noisy, the posterior distribution is fairly wide (grey). The dashed black lines in the bottom panels and the shaded grey section in the top panels indicate the 89% highest posterior density interval (HPDI), i.e. the smallest continuous interval containing 89% of the samples

For an initial demonstration of the software capabilities, where the correct result is known, simulated data was created using the forward model assuming three different growth rate curves: a constant rate, an increasing rate, and a decreasing one. The growth rates for these simulated example data were then inferred using the program. All three were reproduced with sufficient accuracy to be qualitatively easily identifiable (Fig. 2). Quantitatively, some degree of inaccuracy is induced by sampling noise. Note for instance how all of the simulated samples in the constant growth rate case had a greater number of cells than they would on average, leading to a slightly overestimated growth rate. These sources of noise are however to a large degree captured by the width of the posterior distribution. Moreover, this type of error is expected from experiments as well.

### Cell line tests

**Fig. 3:**
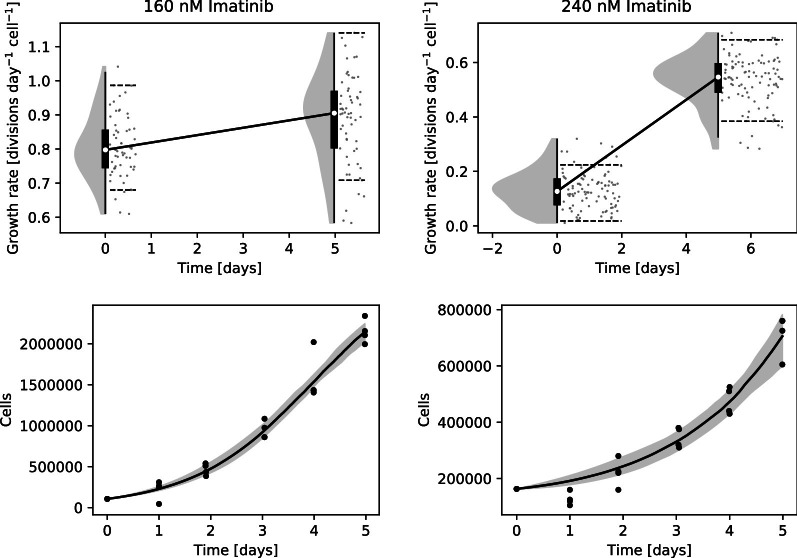
KCL-22 cells growth under the influence of two different concentrations of Imatinib exhibit clearly measurable differences in growth rate. At the higher concentration, the counts after one day were lower than the starting point, which is reflected in the very low deduced initial growth rate. The shaded grey regions indicate the posterior distribution, with grey dots showing individual samples from the posterior distribution. Dashed lines in the raincloud plots indicate the 89% HPDI

Two different experiments with cell lines were carried out to test viability in practice. First, KCL-22 cells were cultured at two different concentrations of imatinib. KCL-22 is a chronic myeloid leukaemia cell line reliant on the onco-protein Bcr-Abl1 for survival, while imatinib is a Bcr-Abl1 inhibitor that effectively inhibits their growth [16]. With that in mind, the imatinib concentrations were selected such that the cells would grow, albeit slowly. The difference in growth rate can be clearly distinguished between the two concentrations (Fig. 3). Under normal conditions KCL-22 cells have a growth rate of about 1 division per day (i.e. population size doubling per day). Imatinib has an inhibitory coefficient, IC50, of 240 nM (as measured by us). Thus, we expect to see growth rates of about 0.8 and 0.5 divisions per day, which is reasonably accurately represented given that IC50 measurements are typically not perfectly reproducible [26].

**Fig. 4:**
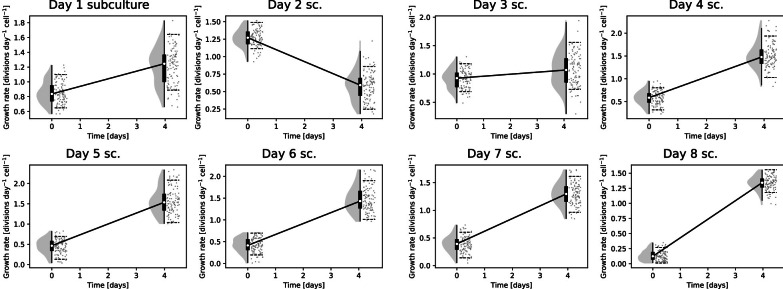
K562 subcultures (sc.) were seeded from a source culture getting more and more overgrown. Initial growth became ever more limited as the cells were exposed to an progressively worse growth environment prior to reseeding, yet they recovered to grow at similar rates after 4 days. The shaded grey regions indicate the posterior distribution, with grey dots showing individual samples from the posterior distribution. Dashed lines in the raincloud plots indicate the 89% HPDI

For the second cell line test, A batch of K562 cells was allowed to overgrow significantly. Each day, a subculture was taken and reseeded down to 1e5 cells/mL (a concentration at which they would normally grow well). These reseeded cultures were then counted every day. Allowing cells to overgrow typically damages their ability to reproduce, thus we expected that the reseeded cultures taken later on would grow slower initially. This was indeed observed; it could be seen that the longer cells had overgrown for, the worse their initial growth had become (Fig. 4). Additionally, the best growth occurred in the second subculture, which is consistent with the standard culturing procedure of splitting every other day. The observed decrease in growth rate in culture number two may have several explanations. First, in practice, as nutrient availability decreases with the increased number of living cells, we may expect a natural decrease in growth rate not accounted for by a simple logistic model of growth. Second, a slight overestimation of the carrying capacity parameter may cause such an artefact as the growing culture does not reach the expected population on time. During the four days for which they were tracked, all the subcultures recovered to grow at similar rates.

The endpoint posterior distributions are generally wider than the starting point. This occurs since when the population size approaches the carrying capacity, changes in the growth rate have an ever smaller effect on the relative change in population size. This is most evident in the extreme example of the final steady state at the carrying capacity, where changes in growth rate have no effects whatsoever. There is essentially less information about the growth rate as the population size approaches the carrying capacity. Note however that measurements of small population sizes are more noisy, which somewhat balances the qualitative effect on the size of the posterior distribution.

Given the width of the posterior distributions in Figs. 3 and 4 the magnitude of changes required to be clearly distinguishable is on the order of a 20% change in growth rate. This should be taken into account when designing experiments intended to use this method. For instance, to observe drug resistance, the drug dose used should inhibit growth of non-resistant cells by at least that much, so as to create an observable difference.

## Conclusions

There are many tools which infer cell-growth parameters from data, but we are not aware of any other tools for inferring a changing growth rate. The methodology is capable of distinguishing both absolute changes in growth rate, and differences specific to initial growth. The magnitude of the effects which could be identified is about a 20% change in growth rate or greater, though this depends on the specifics of the experiment. We did not test a decreasing growth rate on cells, as adaptation is normally positive for evolutionary reasons. Yet, since tracking decreasing rates worked for simulated data, this might be possible as well.

This work enables experiments wherein intentional or spontaneous changes to growth rate are of interest. For instance, it is possible to produce drug resistant cancer cells by culturing them with an inhibitor [16]. In cases where the adaptation happens quickly [15], this method could be used to follow the adaptation process. Another experiment made possible could be to examine the breakdown of some inhibitor, by observing how the growth changes as the concentration drops. This may be of use if the inhibitor is potent enough that the low concentrations are hard to measure directly.

## Materials and methods

KCL-22 and K562 cells were kindly provided by Prof. Leif Stenke (Karolinska Institutet, Solna, Sweden). They were cultured in $37^{\circ }\hbox {C}$, 5% CO$_2$ in RPMI1640 (Gibco, with Glutamax) with 10% HI-FBS (Gibco) and 1% PenStrep (Gibco). Cell counting was performed using trypan blue exclusion on a LUNA-2 cell counter (Logos Biosystems).

### KCL-22 with inhibitors

KCL-22 cells were grown in under either 240 or 160 nM of imatinib, each in quadruplicate, in a 24 well plate. Cultures were initiated at 1e5 cells/mL in 1 mL medium, and were counted every day for 6 days.

### K562 overgrowth

K562 cells were seeded to 1e5 cells/mL. Subcultures, diluted to 1e5 cells/mL, were taken daily for 8 days. All cultures were counted daily, as above.

## Usage and practical details

The program runs on linux and has a simple command line usage powered by a snakemake [27] workflow that automatically processes input data, runs simulations and compiles an output report. It is also possible to manually run all intermediate steps if desired. As ABC is a computationally heavy approach, the deterministic simulator works on a regular laptop, but the stochastic simulator may require a more powerful machine to finish in a timely manner. Data input is prepared as two files, the first of which is a .csv featuring names, live cell counts, data collection times, and optionally information about the steps undertaken during sampling and possible subsequent dilution of the culture. These sampling and dilutions steps are used for simulating sampling noise. The second input file is a .toml file (a common human–readable config–file format) detailing simulation parameters. This file typically only requires a few parameters tuned from defaults. Most importantly: (1) The carrying capacity, i.e. the greatest population size the culture will reach if left alone. (2) How the named data should be grouped; grouping can be relevant if either several measurements are carried out on the same population, or if several populations are expected to behave similarly and one intends to elucidate the overall trend. Detailed annotated examples using the data from the tests below are available in the github repository. Figures 2, 3 and 4 were produced by the program, with minor additions made for clarity.

### Usage example

Consider the following minimal example of cells growing logistically. Suppose we have cells growing in 1 mL of medium, which, if left alone, would reach a total of 3e6 cells (i.e. the carrying capacity). Suppose further we then recorded the following observations (Table 1) which, while presented here as a table for ease of reading, would be provided to the program as a regular .csv file. The measurements were done by first taking a $10~\upmu \hbox {L}$ sample, mixing it with an equal volume of trypan blue dye, transferring the mixture to a haemocytometer, and finally counting the number of living cells in a $0.4~\upmu \hbox {L}$ volume. In Table 1 the name column indicates that we are following one particular cell culture. The time is the time, in days, when the count was made (relative to the first one). count is self-explanatory, whereas sample1 is the fraction of the total population taken in the first sampling step (i.e. the $10~\upmu \hbox {L}$ sample), and sample2 is the fraction of the first sample counted in the second step, ignoring the dilution (as it does not change the number of cells in the sample). Note that $0.4~\upmu \hbox {L}$ in a 1:1 dilution is $0.2~\upmu \hbox {L}$ without dilution, and $10~\upmu \hbox {L} \cdot 0.02 = 0.2~\upmu \hbox {L}$

**Table 1:** Table of observations for the minimal example

Name	Time [days]	Count	Sample1	Sample2
Minimal	0.0	19	0.01	0.02
Minimal	2.0	105	0.01	0.02
Minimal	4.0	403	0.01	0.02
Minimal	6.0	529	0.01	0.02
Minimal	8.0	591	0.01	0.02

The units in brackets would not be part of the input .csv

We run the program using the .toml file with parameters in Listing 1. Note that anything following a “#” are comments with no effect. We are assuming that the growth rate is somewhere between 0.01 and 3.0 divisions per cell and day, and that the growth rate function can be described as a piecewise linear function with either one (constant), two (linearly changing with time) or three (linearly changing with a central breakpoint) control points. Furthermore, we are running four simulations in parallel using the deterministic bernoulli equation simulator (Eq. 2). Finally, each individual count is assumed to be wrong 5% of the time (under a normal distribution approximation of the underlying binomial distribution).
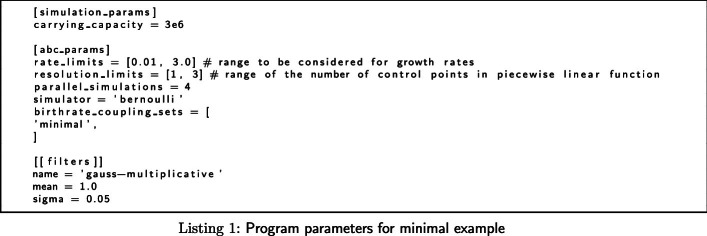


Using data/minimal.csv and data/minimal.toml as input, the snakemake workflow automatically is used to run all simulation steps by calling snakemake results/minimal.pdf results/minimal.fit.csv which yields plots and a table of the inferred growth rates.

## Availability and requirements

- Project name: ratrack
- Project home page: https://github.com/Sandalmoth/ratrack
- Operating system: Linux
- Programming language: Python3 and C++
- Other requirements: Python 3.6 or higher, pyABC 9.14 or higher, snakemake 4.4 or higher. A complete list is provided as a conda environment file available on the project homepage.
- License: zlib (permissive open source)
- Any restrictions to use by non-academics: Not applicable.

## Supplementary information

**Additional file 1: Figure S1.** Cell count timelines collected from K562 cells.

## Data Availability

The datasets supporting the conclusions of this article are included within the article (and its additional files). Simulation code (and datasets) are available at github.com/Sandalmoth/ratrack.

## Abbreviations

PDT: Population doubling time
HPDI: Highest posterior density interval
HI-FBS: Heat-inactivated fetal bovine serum
ABC: Approximate bayesian computation
SMC: Sequential Monte-Carlo
IC50: 50% inhibitory coefficient
