# Supplementary material for: Inferring time-dependent population growth rates in cell cultures undergoing adaptation
Source: BMC Bioinformatics. 2020 Dec 17;21:583. doi: 10.1186/s12859-020-03887-7 (PMC7745411; doi:10.1186/s12859-020-03887-7)
Supplement: Supplementary file 1 — Additional file 1: Figure S1. Cell count timelines collected from K562 cells. [file 12859_2020_3887_MOESM1_ESM.pdf]

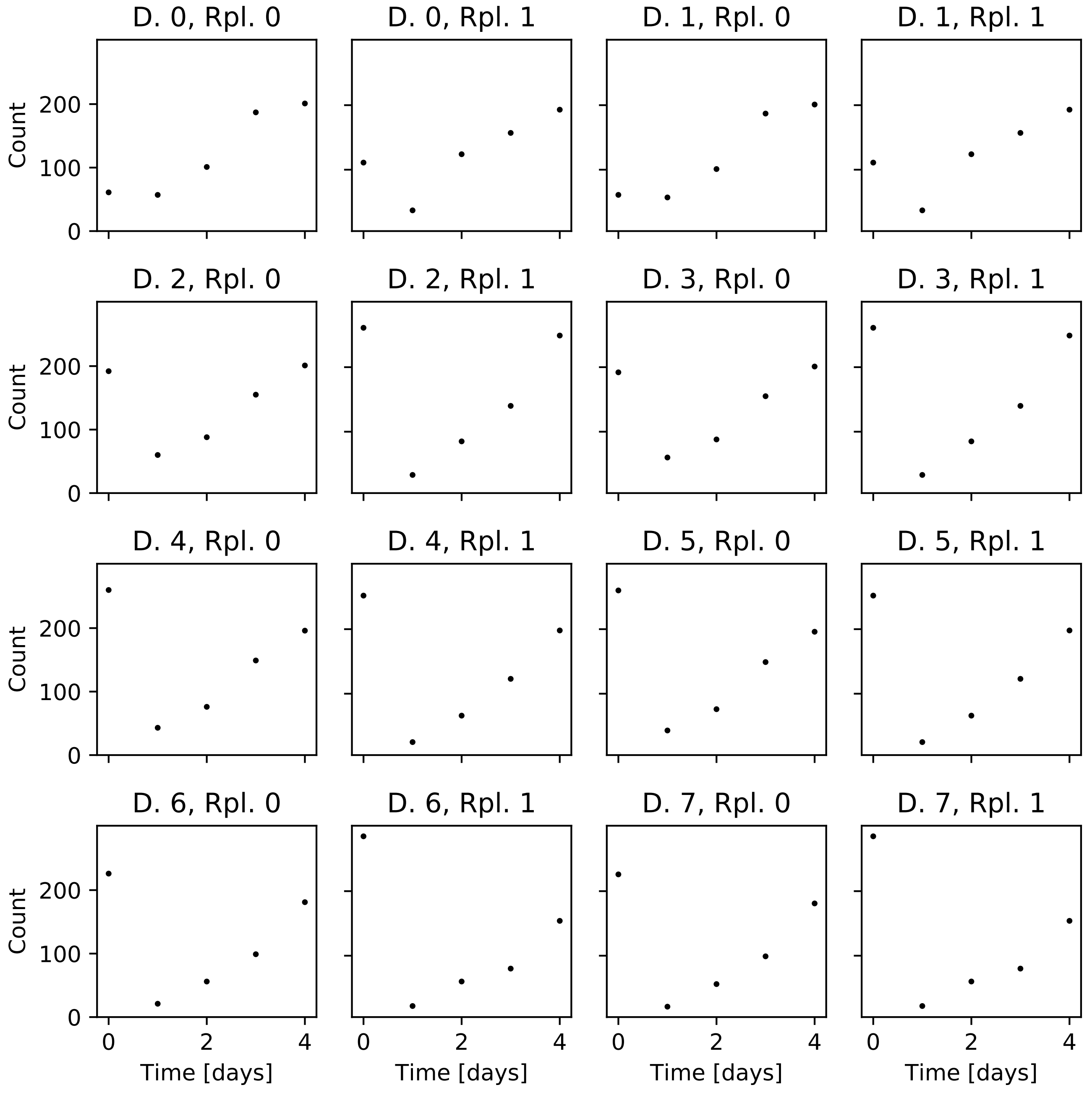

D. 0, Rpl. 0
D. 0, Rpl. 1
D. 1, Rpl. 0
D. 1, Rpl. 1
D. 2, Rpl. 0
D. 2, Rpl. 1
D. 3, Rpl. 0
D. 3, Rpl. 1
D. 4, Rpl. 0
D. 4, Rpl. 1
D. 5, Rpl. 0
D. 5, Rpl. 1
D. 6, Rpl. 0
D. 6, Rpl. 1
D. 7, Rpl. 0
D. 7, Rpl. 1
Count
0
100
200
Time [days]
0
2
4
